# The Mediating Role of Resilience and Electronic Health Literacy in the Relationship Between Pandemic Fatigue and Adherence to Preventive Behaviours Against COVID-19

**DOI:** 10.7759/cureus.29553

**Published:** 2022-09-25

**Authors:** Noha S Hassanien, Abdu M Adawi, Turki A Alzahrani, Essa A Adawi

**Affiliations:** 1 Biostatistics, Alexandria University, Alexandria, EGY; 2 Preventive Medicine, Ministry of Health Saudi Arabia, Riyadh, SAU; 3 Preventive Medicine, Ministry of Health Saudi Arabia, King Abdulaziz Hospital, Taif, SAU; 4 Surgery, Jazan University, Jazan, SAU

**Keywords:** pandemic fatigue, structural equation model, health literacy, resilience, covid-19

## Abstract

Introduction: There is emerging literature on the decline in adherence to preventive measures against the COVID-19 pandemic, a phenomenon of pandemic fatigue (PF). However, academics and policymakers have debated its existence and consequences. We conducted this study to explore this phenomenon, its existence, determinants, and relation to adherence to COVID-19 preventive measures, and the mediating role of resilience, fear of COVID-19, and electronic health literacy about COVID-19 in this relationship.

Methods: This cross-sectional online study was conducted from April to June 2021 using a convenience sample of 650 Saudi adults from all regions of Saudi Arabia using a reliable questionnaire. A structural equation model (SEM) was used for mediation analysis.

Results: The results revealed a moderate level of PF among Saudi adults. Younger patients experienced more PF. Fear of COVID-19 had a non-significant (p=0.127) effect on PF. SEM analysis revealed that both resilience and electronic health literacy significantly (p=0.000) mediated the relationship between PF and adherence to preventive measures, and acted as protective factors. In conclusion, there is evidence that PF exists and negatively affects adherence to preventive measures.

Conclusion: Policymakers should apply evidence-based programs to increase public resilience, particularly targeting young adults, as the results of the current study shed light on its protective mediating role against PF. Increasing electronic health literacy is an effective strategy for preventing PF through an increase in the perceived effectiveness of preventive measures.

## Introduction

The COVID-19 pandemic mandates the application of various restrictions and infection control precautions to prevent the spread of the virus, such as stay-at-home orders, curfews, isolation, social distancing, mandatory hand hygiene in public places, and the obligation to wear face masks. Despite the wide coverage of the COVID-19 vaccine, there is still a need for non-pharmaceutical preventive measures owing to the emergence of new variants which lead to the occurrence of several waves of COVID-19 [1]. The prolongation of this pandemic has had a negative impact on the mental health of the population and increased anxiety and psychological distress, as reported in a recently published meta-analysis [2]. Similar results have been reported as an increase in mental disorders and anxiety during the COVID-19 pandemic in Saudi Arabia [3-5]. In addition, continuous exposure to the flood of information about cases and deaths related to this pandemic has resulted in emotional exhaustion. Consequently, a natural psychological response called pandemic fatigue (PF) has emerged [6,7]. The World Health Organization (WHO) has warned that there is a decline in adherence to preventive measures globally, which may be attributed to this phenomenon.

PF is defined as physical and mental exhaustion and a lack of motivation to follow the recommended protective measures resulting from the application of several restrictions over a long period. It is also associated with a loss of interest in seeking more information regarding COVID-19 [7,8]. However, academics and policymakers have raised a debate about this phenomenon and deflated its existence and effect. In contrast, other scholars expressed their concern about PF and its relation to the wane of adoption of preventive measures against COVID-19 among the public that, in turn, will result in an increase in cases and deaths, posing a public health risk [9-11]. PF is a progressive process that increases over time, which is a challenge in combatting COVID-19 because its surge may result in the prolongation of the pandemic and even lockdown with its serious impacts on the socio-economic aspect of the population and mental well-being. Studies have also reported that PF may cause more psychological distress and, affect sleep quality and productivity at work [7,9]. WHO suggests that to combat PF, the government should understand this phenomenon and its determinants so that tailored policies and interventions can be applied to curb it [6]. Several factors may interact and result in fatigue and demotivation. According to the health belief model (HBM) constructs [12], which is the most commonly used model to interpret the factors influencing the behaviour of a community regarding health threats, the factors which influence individual behaviours are perceived severity, perceived susceptibility, perceived benefits, and barriers. Previous studies have determined other contributing factors for the development of PF, such as social, environmental, and personal factors [13], over-flooding of information from media [14], and emotions such as fear [13]. Evidence regarding the specific interactions and mediating mechanisms of these factors is limited. Recent studies related to PF have mainly addressed its existence and effects among special groups such as healthcare workers and university students. However, few studies have focused on the general public. Study results among healthcare workers revealed that support either, social or psychological, decreases PF [14,15]. A study conducted by Labrague found that university students suffered from PF, and the main contributing factor was fear of COVID-19 [16]. Resilience is seen as a protective behaviour against stressful events in psychology, allowing individuals to bounce back to their normal condition following stressful situations, and it is completely different from mental well-being [17,18]. During the pandemic, resilience was associated with a reduction in negative psychological consequences and improved mental health, as reported in previous studies [19,20]. Thus, strengthening resilience may help combat PF. Another protective coping strategy to fight PF, which has been reported in previous studies, is electronic health literacy. This is defined as the ability to find and evaluate health-related information on the Internet which is intended to have a favourable impact on people’s motivation to adopt preventive behaviours [21]. Some studies have considered it a social vaccine to counteract the effects of the pandemic and overwhelming information [21-23]. According to the HBM, which is our theoretical framework in this study, increased perceived benefits and decreased perceived barriers lead to adherence to preventive measures against COVID-19, and this can be achieved by good health literacy [24,25]. Although several studies have been conducted on resilience and electronic health literacy during the COVID-19 pandemic, the exact mechanism of their effects on PF and adherence to preventive measures remains unclear.

Despite several studies being conducted to examine PF and its associated factors, some of them did not use a specific tool for its assessment during the COVID-19 pandemic; Nitschke et al., in Austria, used the global fatigue assessment tool and reported a negative association between social engagement, stress, fear, and PF. Fear of COVID-19 may motivate people to adhere to preventive measures; however, due to the uncertainty about the end of this pandemic, mental exhaustion leads to higher PF [26].

To our knowledge, no study has investigated PF in Saudi Arabia. There is also insufficient evidence on the variables that contribute to the evolution of this phenomenon, or the factors that could alleviate its consequences. We undertook our research to examine the current situation of PF among Saudi adults, its drivers, its implications on the deployment of preventive actions, and the influence of personal resilience, fear of COVID-19, and electronic health literacy about COVID-19 in the mediation of PF. Our study incorporated both HBM and electronic health literacy skills to explore the mediators of PF using structural equation model (SEM) analysis, a robust statistical model used in medication analysis [27]. In this multiple mediation model, we hypothesized that there is a negative impact of PF on people’s adherence to protective measures (H1). Personal resilience mediated the relationship between PF and adherence to protective measures (H2). Coronavirus fear mediated the relationship between PF and adherence to protective measures (H3). E-health literacy mediated the relationship between PF and adherence to protective measures (H4).

## Materials and methods

Study design, sampling, and data collection

An online cross-sectional study was conducted by posting a questionnaire via all social media platforms to all adults in different provenance of Saudi Arabia. The data were collected from April to June 2021.

We obtained institutional ethical approval from King Abdelaziz City for Science and Technology, Institutional Review Board (HAP-02-T-067). Epi Info™7 software (Centers for Disease Control and Prevention (CDC), Atlanta, Georgia, USA) was used to calculate the sample size using a margin of error of 5% and confidence level of 95%, assuming that the level of PF and adherence to preventive behaviour is 50% among Saudi adults. The minimum sample size was 384 individuals. A convenience sample was used to recruit participants without any exclusion criteria. After a thorough review of previous studies on the same topic (6-8), a questionnaire was created. It was first written in English, then translated and back-translated (English-Arabic) by two multilingual specialists, with the questionnaire being changed in accordance with their recommendations. In a pilot study that included 50 participants, reliability was tested, and the necessary changes were made. The questionnaire consisted of the following sections:

Socio-Demographic and Health Data

These included sex, age, education, employment, region of residence, marital status, monthly household income in Saudi Riyal, history of COVID-19 infection of the participants or any of their relatives or friends, and self-reported history of chronic diseases.

Electronic Health Literacy Scale About COVID-19

The Arabic version of the EHL scale was used [28]. It includes eight questions with a Cronbach’s alpha of 0.939, indicating excellent reliability. In addition, the sources of knowledge and whether they are official sources, such as Ministry of Health (MoH) channels, were inquired about.

Perceived Risk

Two questions were used to assess risk perception: one related to the perceived severity of COVID-19 (Do you think COVID-19 is dangerous? Yes/no) and the other related to perceived susceptibility (Do you think that you are susceptible to COVID-19? Never/poor possibility/intermediate possibility/strong possibility.

Perceived Benefits

This was assessed by one question (Do you think that compliance with preventive measures is beneficial for the prevention of COVID-19 infection? Yes/no)

Fear of Coronavirus-19 Scale

The validated Arabic version of the Fear of COVID‑19 Scale (FCV‑19S) was used to measure the fear levels of COVID‑19 during the past 14 days [29]. It included seven questions, and participants were asked to rate their responses on a 5-point Likert scale ranging from 1 (totally disagree) to 5 (totally agree). The total score is obtained by the summation of all items and ranges from seven to 35. The highest score was for severe fear of COVID‑19. The Cronbach’s alpha in this study was acceptable (0.699).

Adherence to Protective Behaviours

Adherence to protective behaviors toward COVID-19 was measured via a composite score of 14 items that covered self-reported protective behaviours in three aspects: avoidant, preventive, and management of disease behaviorus in the past 14 days. Avoidant behaviours include social distancing, such as avoiding crowds or hugging and kissing people, unnecessary travel, and social meetings. Preventive behaviours include hand-washing, wearing of masks outside home, and cough etiquette. Disease management includes seeking medical advice and self-isolation if COVID-19 is suspected. The Cronbach’s alpha was very good (0.850), indicating good internal consistency. The responses were then summed across the items to generate total scores.

Brief Resilience Scale (BRS)

Resilience was assessed with the validated BRS [30]. This included six items to be answered on a 5-point Likert scale ranging from “totally disagree” to “totally agree”. Half of the items were reverse-worded and coded [19,30]. Cronbach’s alpha showed good internal consistency (0.835).

PF Scale

The PF questionnaire was used to assess mental and physical fatigue among participants which consisted of six items rated using a Likert scale (1 for ‘strongly disagree to 5 for strongly agree). Higher scores on the scale indicate higher levels of PF [31]. Cronbach’s alpha was very good (0.873).

Data analysis

The data was analyzed using SPSS, version 25.0 (IBM Corp., Armonk, NY). The categorical variables were presented as frequency and percentages. The normality of quantitative variables was assessed by the Kolmogorov-Smirnov test. A bivariate analysis was conducted to determine the factors that affect the level of PF and adoption of protective behaviors using the Man-Whitney test (two groups) and the Kruskal-Wallis test (more than two groups) that is followed by post-hoc testing using the Bonferroni test. Spearman correlation was used to assess the correlation between e-health literacy score, fear of COVID-19 score, adherence to protective behaviors scores, and resilience. All significant variables (p< 0.05) were included in the multiple linear regression analysis models where the dependent variable was PF after testing of the regression assumptions. The SEM model was constructed to explore the mediation effect of resilience and e-health literacy in the relation between PF and adoption of protective behaviour using analysis of moment structures (AMOS) Graphics 22 software. The validity of the SEM model was assessed by multiple indices: comparative fit index (CFI), adjusted goodness-of-fit index (AGFI), normal fit index (NFI), the model was considered fit with values greater than 0.90 also, root mean square error approximation (RMSEA) was used to assess the model fit (value of less than 0.05 indicate good fitness) [32]. To test our hypothesis that personal resilience and e-health literacy mediate the relationship between PF and adherence to protective behaviour against COVID-19, the SEM was conducted to prove this hypothesis (H2 and H4). We deflated H3 as fear had a non-significant correlation with PF. We used the maximum likelihood estimation method and 200 bootstrapping technique.

## Results

Descriptive statistics

A total of 650 participants responded to the questionnaire and their socio-demographic characteristics (Table 1). About 46% of participants were in the age group from 30 to less than 50 years old and the median age for participants was 35 years. The majority of them (61.2%) were males. Most of them (76.3%) were married and 64.5% of them had a university education. About 44% of them had a monthly income of 15 or more thousand Saudi Riyals.

**Table 1:** Socio-demographic characteristics of participants

Characteristics	N= 650
Age in years	No. (%)
18-	197 (30.3)
30-	299 (46.0)
50+	154 (23.7)
Mean ± SD (median, IQR)	38.4± 12 (35, 20)
Gender	No. (%)
Male	398 (61.2)
Female	252 (38.8)
Marital status	No. (%)
Single	135 (20.8)
Married	496 (76.3)
Divorced or widow	19 (2.9)
Education level	No. (%)
Primary or intermediate	5 (0.8)
Secondary	93 (14.3)
University	419 (64.5)
Above university	133 (20.5)
Employment status	No. (%)
Unemployed or housewife	72 (11.1)
Employed (governmental and private)	444 (68.3)
Student	57 (8.8)
Retired	77(11.8)
Income in Saudi Riyals	No. (%)
< 3000	49 (7.5)
3000-	149 (22.9)
9000-	169 (26.0)
15000+	283 (43.6)
Region	No. (%)
Central	73 (11.2)
Western	234 (36.0)
Eastern	70 (10.8)
Southern	221 (34.0)
Northern	52 (8.0)

Table 2 showed that only 17.8% were smokers, 28.6% had chronic diseases, 2.6% had a positive history of COVID-19 infection, 17.5% had a history of contact with COVID-19 patient, 48.6% of them had a relative or friend infected with COVID-19, and 65.8% were vaccinated against COVID-19. About 35% of participants perceived that they have the intermediate possibility for COVID-19 infection and 24.5% strong possibility. The majority (65%) perceived the severity of COVID-19 infection. About 71% perceived effectiveness of preventive measures toward COVID-19. Almost 80% sought information about COVID-19 from official sites.

**Table 2:** Distribution of smoking status, medical history and COVID-19 related data among participants

Characteristics	N= 650
Smoking	No. (%)
No	534 (82.2)
Yes	116 (17.8)
History of chronic diseases	No. (%)
No	464 (71.4)
Yes	186 (28.6)
History of past infection with COVID-19	No. (%)
No	633 (97.4)
Yes	17 (2.6)
History of contact with COVID-19 patient	No. (%)
No	536 (82.5)
Yes	114 (17.5)
Have a relative or friend infected with COVID-19	No. (%)
No	334 (51.4)
Yes	316 (48.6)
Presence of chronic diseases	No. (%)
No	464 (71.4)
Yes	186 (28.6)
COVID-19 vaccine	No. (%)
No	222 (34.2)
Yes	428 (65.8)
Perceived susceptibility	No. (%)
Never	108 (16.6)
Poor possibility	154 (23.7)
Intermediate possibility	229 (35.2)
Strong possibility	159 (24.5)
Perceived Severity No.	No. (%)
No	231 (35.5)
Yes	419 (64.5)
Perceived effectiveness of preventive measures	No. (%)
Not, effective	46 (7.1)
Yes, effective	460 (70.8)
May be	144 (22.2)
Seeking information about COVID-19 from official sites	No. (%)
No	127 (19.5)
Yes	523 (80.5)

Descriptive statistics and correlation between all study measures

The correlation matrix revealed that PF had a highly significant (p=0.0000) inverse correlation with the adoption of preventive behavior; e-health literacy had a significant weak inverse correlation with fear of COVID-19, and PF (p=0.007, p=0.000 respectively). Resilience had a significant inverse correlation with both fear of COVID-19 and PF.

The mean health literacy score is 19.8 (SD=8.7), mean score of adoption of preventive behavior is 51.8 (SD=8.7), both are above the midpoint. Both fear of COVID-19 and PF showed average scores above midpoint (18.6 and 17.8 respectively) while personal resilience average score was 14.5 (Table 3).

**Table 3:** Descriptive statistics and correlation coefficients of all studied variables

	Mean ± SD	Median (IQR)	E-Health literacy	Preventive behavior	Coronavirus Fear	Pandemic fatigue
E-Health literacy	19.7 ± 8.6	17(13 -25)				
Preventive behavior	51.8 ± 8.7	53 (51-57)	0.395 (p = 0. 000* )			
Coronavirus Fear	18.6 ± 4.9	19 (15-23)	-0.106 (p = 0.007* )	-0.018 (p = 0.644)		
Pandemic fatigue	17.8 ± 7.0	16 (13-21)	-0.651 (p =0.000* )	-0.519 (p = 0.000* )	0.060 (p =0.127)	
Resilience	14.5 ± 6.4	12 (10-18)	0.847 (p = 0. 000* )	0.381 (p = 0.000* )	-.120 (p = 0.002* )	-0.627 (p = 0.000* )

Determinants of PF and adoption of protective behavior among participants

Regarding the socio-demographic factors that were associated with PF, only age was significantly associated with PF (p=0.005). The post hoc test showed that the mean score among age from 18 to less than 30 years had the higher PF score than older age categories. In contrast, the younger age group was significantly lower in average scores for adherence to protective measures. Also, gender had a highly significant (p=0.000) effect on preventive behaviour where females had a significantly higher average score than males (53.2 VS 50.9). Single individuals had a significantly (p=0.004) lower preventive behaviour score than those who were married or divorced or widowed (50.8, 51.9, and 56.7 respectively). Unemployed or housewife had a significantly (p=0.049) lower average score than other employment categories (50.9 ± 8.5) as shown in Table 4.

**Table 4:** Socio-demographic data associated with pandemic fatigue and protective behavior among participants

Characteristics	pandemic fatigue Mean ±SD (median, IQR)	P-value	Protective behavior Mean ±SD (median, IQR)	P-value
Age in years				
18-	19.2 ± 7.4 (17, 16)	0.005^*^	50.3 ± 9.8 (52, 7)	0.007^*^
30-	17.4 ± 6.7 (16, 8)		52.6 ± 7.3 (53, 7)	
50+	16.8 ± 6.7 (15, 7)		52.2 ± 9.7 (54, 7)	
Correlation Coefficient	-0.113	0.004^*^	0.116	0.003^*^
Gender				
Male	18.1± 6.9 (16,9)	0.055	50.9 ± 9.1 (51.9, 7)	0.000^*^
Female	17.1±7.1 (15, 8)		53.2 ± 8.0 (54, 7)	
Marital status				
Single	17.9 ± 7.2 (17, 16)		50.8 ± 10.1 (52, 9)	0.004^*^
Married	17.8 ±6.9 (16, 9)	0.347	51.9 ± 10.1 (53, 6.8)	
Divorced or widow	14.9 ±5.1 (15, 7)		56.7 ± 4.7 (56, 5)	
Education level				
Primary or intermediate	21 ± 9.5 (20, 17.5)		56.6 ±6.8 (57, 13)	
Secondary	18.6 ±7.5(17,14.5)		50.6 ± 11.5 (54, 7)	0.448
University	18.0 ± 7.1 (16, 9)	0.080	51.7 ± 8.5 (53, 6)	
Above university	16.3 ±6.1 (15, 7)		52.7 ±7.1 (53, 7)	
Employment status				
Unemployed or housewife	19.6 ± 7.6 (17,15.7)		50.9 ± 8.5 (52,6.7)	0.049^*^
Employed	17.8 ± 7.0 (16, 8)		51.7 ± 8.6 (53, 6)	
Student	16.8 ± 6.8 (16, 7)	0.085	52.1 ±9.2 (54, 6)	
Retired	16.6 ± 6.2 (15, 6)		52.8 ± 9.5 (55, 6)	
Income in SR				
< 3000	19.7 ± 7.5 (18, 14)		50.5 ± 12.2 (54, 8)	
3000-	17.5 ±7.3 (16, 8.5)	0.262	52.0± 9.3 (53, 8.5)	0.161
9000-	17.6 ± 6.8 (16, 8)		52.8 ±8.2 (53, 8)	
15000+	17.7 ± 6.7 (16, 7)		51.3 ±8.1 (52, 7)	

Both perceived severity and perceived susceptibility had no significant (p > 0.05) association with PF but only perceived severity of COVID-19 is significantly associated with the adoption of protective behaviour. Participants who perceived the effectiveness of protective measures had significantly lower PF and a significantly higher score for adherence to preventive measures (Table 5).

**Table 5:** Health data associated with pandemic fatigue and protective behavior among participants

Characteristics	Pandemic fatigue Mean ±SD (median, IQR)	P- Value	Protective behavior Mean ±SD (median, IQR)	P- Value
Smoking				
No	17.7 ± 6.9 (16, 7)	0.753	51.9± 8.8 (53, 7)	0.666
Yes	18.2 ± 7.3 (16, 12.7)		51.5 ± 8.5 (53, 6)	
History of chronic diseases				
No	17.8 ± 7.1 (16, 8)	0.974	51.8± 8.3 (52, 6.7)	0.107
Yes	17.7 ± 6.8(16, 7.3)		51.7 ± 9.8 (54, 7)	
History of past infection with COVID-19				
No	17.7 ± 6.9(16, 8)	0.139	51.8± 8.8 (53, 7)	0.429
Yes	20.5 ± 7.5(18, 16)		51.2 ± 7 (51, 5.5)	
History of contact with COVID-19 patient				
No	17.6 ± 6.8(16, 7)	0.085	52.0± 8.6 (53, 7)	0.095
Yes	18.9 ± 7.3(17, 14.3)		50.8 ± 9.4 (52, 6)	
Have a relative or friend infected with COVID-19				
No	17.4 ± 7.2(15, 9)	0.068	51.8 ± 9.6 (53, 7)	0.783
Yes	18.2 ± 6.8(16.0, 8)		51.9 ± 7.7 (53, 6.3)	
Presence of chronic diseases				
No	17.8 ± 7.1 (16, 8)	0.974	51.9± 8.8 (53, 7)	0.107
Yes	17.7 ± 6.9(16, 7.3)		51.5 ± 8.5 (53, 6)	
COVID-19 vaccine				
No	17.6 ± 7.2 (16, 8.3)	0.759	50.8±8.1 (52, 4)	0.000^*^
Yes	17.9 ± 6.9 (16, 8)		52.3±9.1 (54, 8)	
Perceived severity				
No	17.8 ± 7.1 (16, 8)	0.947	50.8± 7.9 (52, 4)	0.000^*^
Yes	17.7 ± 6.9 (16, 8)		52.3±9.2 (54, 8)	
Perceived susceptibility				
Never	17.0 ± 7.2 (15, 9)	0.070	51.9± 8.9 (54, 7)	0.530
Poor possibility	18.3 ± 7.2 (16, 13)		51.5± 9.5 (52, 6)	
intermediate possibility	18.3 ± 6.9 (17, 8)		51.2± 8.2 (53, 6.5)	
Strong possibility	17.0 ± 6.6 (15,7)		52± 8.8 (54, 7)	
Perceived effectiveness of protective measures				
No	22.3 ± 8.3 (22, 16)	0.001^*^	37.7± 16.6 (44, 26)	0.000^*^
Yes	17.3 ± 6.6 (16, 7)		52.9± 6.7 (54, 7)	
May be	17.9± 7.2 (16, 10.5)		52.6± 7.0 (53, 6)	
Seeking information about COVID-19 from official sites				
No	18.1 ± 8.2 (16, 18)	0.679	52.6±9 (54, 8)	0.227
Yes	17.7 ± 6.6 (16, 7)		51.6 ± 8.7 (53, 7)	

Table 6 shows the results of the multiple linear regression model which revealed that the significant predictors of PF, by order of magnitude, are e-health literacy (β= -0.402), resilience (β= - 0.188), and perceived effectiveness of protective measures (β= -0.069). All included variables lead to a decline in a PF score and explain about 32.1% in the variation of PF score.

**Table 6:** Multiple linear regression for significant predictors of pandemic fatigue

Predictors	Pandemic fatigue score				
	β	SE	P-value	95% CI	
				LL	UL
Perceived effectiveness of protective measures	-0.069	.438	0.035	-1.7	-0.066
Health literacy about COVID-19	-0.402	.041	< 0.001	-0.409	-0.248
Resilience	-0.188	. 055	< 0.001	-0.313	-0.096
Model parameters	F=77, P= < 0.001 * R^2 ^ = 0.321				

Mediation analysis

**Figure 1:**
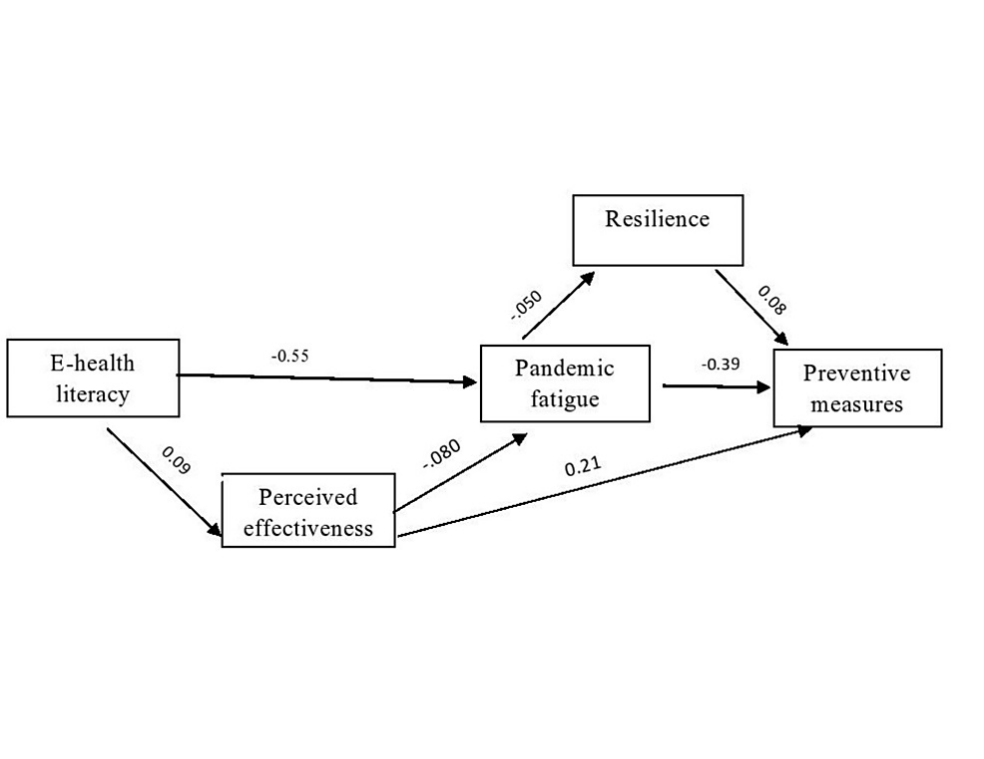
Structural equation model (SEM) analysis of the mediating effect of resilience and e-health literacy on the association between pandemic fatigue and protective behaviors toward COVID-19 pandemic

Figure 1 revealed that resilience was a significant mediator in a hypothesized relationship and total effect (both direct and indirect effects) had a p-value of 0.000. Resilience had a significant negative effect on PF (β= -.50, p< 0.05), significant positive effect on preventive behaviour (β= .08, p< 0.05) and PF had a significant negative effect on preventive behaviors (β= -.39, p< 0.05). E-health literacy was a significant mediator in a hypothesized relationship and its total effect (both direct and indirect effects) had a p-value of 0.000. It had a significant direct negative effect on PF (β= -.55, p< 0.05), and indirect effect through increase in perceived effectiveness of preventive measures (β= .09, p< 0.05). Perceived effectiveness of preventive measures had a significant positive effect on preventive behaviour (β= 0.21, p< 0.05). The final model showed a good fit indices (χ 2 = .524 with a p-value 0.469, CFI=0.999, AGFI=0.994, NFI=0.997; and RMSEA=0.000).

## Discussion

Following WHO's announcement of the COVID-19 pandemic, Saudi Arabian authorities imposed a state-wide mandatory lockdown, curfew, and travel restrictions to combat the spread of the pandemic [33]. These tactics are effective in slowing the propagation of the epidemic; however, they have a variety of negative effects on public mental health. To bridge this gap in the literature, we conducted this study to determine the presence of PF, one of the documented repercussions of the chronicity of pandemic. This issue is linked to a lack of motivation to adopt COVID-19 prevention measures in a psychological phenomenon called PF [6]. This study aimed to investigate the extent to which the general public experienced PF, and the mediating effect of fear of coronavirus, resilience, and health literacy. We expected that these variables would significantly explain the variation in the PF scores.

PF has been studied in various populations. To our knowledge, this is the first study in Saudi Arabia to examine the extent of the PF experienced by the public. The average score in the Saudi society was 17.8 (SD=7) out of a total of 30, indicating a moderate level of PF. However, given the scarcity of studies on this phenomenon and the use of diverse measures to quantify it in available studies, we are unable to compare our findings to those of other researchers. Our findings matched those of prior studies conducted among the general public that found a rise in physical and psychological tiredness and a loss of interest to comply with the usual activity amid the COVID-19 pandemic [34,35] and was less than the average PF score reported by a study conducted by Kim et al. (22.3 ±7.2) [36] and that conducted by Labrague et al. among university students using the same scale (31.5 ±6.9) [16].

This study explored the factors associated with PF. None of the socio-demographic and personal factors were significantly associated with PF, except for age. The younger age group experienced more PF. This could be explained by the fact that young adults are more engaged in social activities, and so they are more likely to be psychologically affected by the restriction of activity during the COVID-19 pandemic. Furthermore, younger people are more exposed to the social media flood of information related to the pandemic, which may cause psychological overwhelming. In addition to the documented results of other studies, older age was linked to a less negative response to the COVID-19 pandemic [36-38]. Therefore, more efforts should be directed to support the mental health of young adults to combat PF. Some of the supportive strategies reported in a meta-analysis conducted by Li et al. [39] were digital and online-based wellness programs, boot camps for coping strategies, and virtual exercise and mindfulness therapy. In addition, virtual clinics for online mental health can be provided during the pandemic.

In our study, there was no gender discrepancy in PF, which is consistent with a study conducted among Australians that found that gender was not substantially associated with PF [37]. In contrast, the female gender was a significant predictor of the PF phenomenon according to a study conducted in China; these variances may be related to cultural differences, as females are more likely to be more expressive of their feelings related to stress and mental exhaustion [40]. In addition, studies have reported a significant association between PF and low socioeconomic status [41,42].

Risk perception of COVID-19 had no significant effect on PF. In contrast, the perceived effectiveness of protective measures significantly lowers PF. This could explain the moderate amount of PF in our study, where the majority of participants (71%) thought that preventive measures were beneficial in preventing COVID-19; therefore, they had less PF. Furthermore, this indicated the success of the communication strategies of the Saudi MoH with the public, which fosters the perception of benefits of the use of protective measures against COVID-19. This result sheds light on the perceptions that should be tackled by interventions against PF, in which the benefits of preventive measures should be emphasised.

According to our research, Saudi Arabia has an above-average score fidelity for preventive activities. However, some socio-demographic factors have an impact on this adherence. Males, as well as younger and unmarried people, were less committed to preventive actions than females. These findings are consistent with a variety of studies conducted in various nations worldwide [43,44].

Individuals who perceived COVID-19 as severe had a higher rate of adherence to preventive interventions, which is consistent with an earlier study [45]. Surprisingly, people who received the COVID-19 vaccine were more likely to follow preventive measures and the vaccine had no link to PF. It was expected that vaccinated individuals would be less adherent and more liable to PF because they perceive themselves as low-risk. This could be explained by the good health literacy of participants about COVID-19 infection and the possibility of being infected despite vaccine intake, as illustrated by the continuous health education messages related to maintaining adherence to protective behaviours even after vaccination and the non-significant association between risk perception and PF. Similar results are being reported from the UK [34].

The high rate of infection and severity of COVID-19 have triggered feelings of fear and panic in the public. Several studies in different countries have demonstrated an elevated level of fear in the communities during the COVID-19 pandemic. According to the findings of this study, the mean COVID-19 fear score was 18.6 (SD=4.8), which is similar to that reported in other countries [10,46,47]. The fear of COVID-19 causes stress and emotional strain, which can lead to an increase in mental exhaustion, resulting in PF. Fear levels among Saudi adults were not high and hence did not affect PF or adherence to preventive measures in our study. It is worth noting that this fear was mitigated by a high degree of personal resilience and health literacy in society because our results showed a significant negative relationship between both e-health literacy and resilience with fear. This makes it critical for policymakers to enhance societal awareness. The results showed that e-health literacy was average, which is in line with the results of other studies [48,49]. Therefore, there is a need to increase electronic health literacy among the public.

According to the findings of this research, the significant predictors of PF were e-health literacy, resilience, and perceived effectiveness of preventive activities, in that order, as determined by regression analysis, which explained 32.1% of the variation in the PF score which is considered large effect size [50]. Another study conducted among university students found that the significant predictors associated with a decline in PF score were male gender, higher education, high resilience, and coping skills, which explained 15% of the variation in PF score; therefore our model was stronger. Additionally, there was a significant association between PF and a decline in adherence to COVID-19 prevention measures. According to the structural equation model analysis, both personal resilience and e-health literacy showed a significant mediation effect in the pathway between PF and adherence to preventive measures. This significant mediating relationship can be interpreted as individuals with high resilience and high electronic health literacy scores showing significantly lower PF. Both exhibited a protective role against PF, resulting in greater adherence to protective interventions. This is consistent with other studies that show that resilience and health literacy have a positive impact on people's mental health and health behavior [40,49,51]. This finding has a significant influence on the prevention of PF by increasing public resilience and electronic health literacy. However, there is a lack of evidence regarding the effective interventions that could promote resilience, as reported in a recently published meta-analysis [52], but it suggests the application of some measures such as social, psychological, religious support, entertainment, and social network interactions.

Our results explain the mechanism by which electronic health literacy affects PF, and it is clear that it had an indirect effect by enhancing the perceived effectiveness of preventive measures.

Given that Saudi Arabia has a moderate degree of PF, as time passes, individuals may experience higher levels of exhaustion, affecting their behavioural and cognitive skills. This necessitates the implementation of evidence-based measures to address the problem. Our study sheds light on some of these interventions.

Implications

Policymakers should not focus only on preventive measures against the epidemic by ordering restrictions, but should also consider psychological factors such as PF, as they may order an application for those measures and the public might not comply. This could be obtained through mental services support, especially for young people, to disseminate timely and accurate online information about COVID-19 in relation to protection measures and the scientific evidence about the benefits of preventive measures.

Limitations

We used self-reported instruments to gather data on the study variables, which may be prone to recall or social desirability bias. However, self-reporting is a typical source of information in research amid the COVID-19 pandemic, and we conducted reliability tests of all measures. Although using a convenience sample may limit the generalizability of the study, we distributed the questionnaire over all regions of Saudi Arabia and had an adequate sample size.

## Conclusions

This study adds to the present evidence of the existence of PF and documents its association with a decrease in adherence to protective behaviours during the COVID-19 pandemic among adults in Saudi Arabia. Additionally, both resilience and electronic health literacy reduce the negative effect of PF on adherence to preventive measures against COVID-19. However, the magnitude of the effect of e-health literacy is higher than that of resilience in combating PF. Additionally, resilience and electronic health literacy were negatively associated with fear of COVID-19. Thus, in an attempt to combat PF, policymakers should deliver evidence-based programs focused on enhancing resilience and electronic health literacy among the public, especially among young adults. In addition to assessing interventions that lead to increased population resilience, further intervention research is needed to identify other potentially mediating variables and how to mitigate them.
